# The 2023 Impact of Inflammatory Bowel Disease in Canada: Treatment Landscape

**DOI:** 10.1093/jcag/gwad015

**Published:** 2023-09-05

**Authors:** Sanjay K Murthy, Adam V Weizman, M Ellen Kuenzig, Joseph W Windsor, Gilaad G Kaplan, Eric I Benchimol, Charles N Bernstein, Alain Bitton, Stephanie Coward, Jennifer L Jones, Kate Lee, Juan-Nicolás Peña-Sánchez, Noelle Rohatinsky, Sara Ghandeharian, Nasruddin Sabrie, Sarang Gupta, Gurmun Brar, Rabia Khan, James H B Im, Tal Davis, Jake Weinstein, Joëlle St-Pierre, Roxana Chis, Saketh Meka, Eric Cheah, Quinn Goddard, Julia Gorospe, Jack Kerr, Kayla D Beaudion, Ashley Patel, Sophia Russo, Jonathan Blyth, Stephanie Blyth, Diane Charron-Bishop, Laura E Targownik

**Affiliations:** Department of Medicine, University of Ottawa, Ottawa, Ontario, Canada; The Ottawa Hospital IBD Centre, Ottawa, Ontario, Canada; Division of Gastroenterology and Hepatology, Mount Sinai Hospital, University of Toronto, Toronto, Ontario, Canada; Department of Medicine, Temerty Faculty of Medicine, University of Toronto, Toronto, Ontario, Canada; SickKids Inflammatory Bowel Disease Centre, Division of Gastroenterology, Hepatology, and Nutrition, The Hospital for Sick Children, Toronto, Ontario, Canada; Child Health Evaluative Sciences, SickKids Research Institute, The Hospital for Sick Children, Toronto, Ontario, Canada; Department of Medicine, University of Calgary, Calgary, Alberta, Canada; Department of Community Health Sciences, University of Calgary, Calgary, Alberta, Canada; Department of Medicine, University of Calgary, Calgary, Alberta, Canada; Department of Community Health Sciences, University of Calgary, Calgary, Alberta, Canada; SickKids Inflammatory Bowel Disease Centre, Division of Gastroenterology, Hepatology, and Nutrition, The Hospital for Sick Children, Toronto, Ontario, Canada; Child Health Evaluative Sciences, SickKids Research Institute, The Hospital for Sick Children, Toronto, Ontario, Canada; ICES, Toronto, Ontario, Canada; Department of Paediatrics, Temerty Faculty of Medicine, University of Toronto, Toronto, Ontario, Canada; Institute of Health Policy, Management, and Evaluation, Dalla Lana School of Public Health, University of Toronto, Toronto, Ontario, Canada; Department of Internal Medicine, Max Rady College of Medicine, Rady Faculty of Health Sciences, University of Manitoba, Winnipeg, Manitoba, Canada; University of Manitoba IBD Clinical and Research Centre, Winnipeg, Manitoba, Canada; Division of Gastroenterology and Hepatology, McGill University Health Centre IBD Centre, McGill University, Montréal, Quebec, Canada; Department of Medicine, University of Calgary, Calgary, Alberta, Canada; Department of Community Health Sciences, University of Calgary, Calgary, Alberta, Canada; Departments of Medicine, Clinical Health, and Epidemiology, Dalhousie University, Halifax, Nova Scotia, Canada; Crohn’s and Colitis Canada, Toronto, Ontario, Canada; Department of Community Health and Epidemiology, University of Saskatchewan, Saskatoon, Saskatchewan, Canada; College of Nursing, University of Saskatchewan, Saskatoon, Saskatchewan, Canada; Crohn’s and Colitis Canada, Toronto, Ontario, Canada; Department of Medicine, Temerty Faculty of Medicine, University of Toronto, Toronto, Ontario, Canada; Department of Medicine, Temerty Faculty of Medicine, University of Toronto, Toronto, Ontario, Canada; Department of Medicine, Temerty Faculty of Medicine, University of Toronto, Toronto, Ontario, Canada; SickKids Inflammatory Bowel Disease Centre, Division of Gastroenterology, Hepatology, and Nutrition, The Hospital for Sick Children, Toronto, Ontario, Canada; Child Health Evaluative Sciences, SickKids Research Institute, The Hospital for Sick Children, Toronto, Ontario, Canada; ICES, Toronto, Ontario, Canada; SickKids Inflammatory Bowel Disease Centre, Division of Gastroenterology, Hepatology, and Nutrition, The Hospital for Sick Children, Toronto, Ontario, Canada; Child Health Evaluative Sciences, SickKids Research Institute, The Hospital for Sick Children, Toronto, Ontario, Canada; SickKids Inflammatory Bowel Disease Centre, Division of Gastroenterology, Hepatology, and Nutrition, The Hospital for Sick Children, Toronto, Ontario, Canada; Child Health Evaluative Sciences, SickKids Research Institute, The Hospital for Sick Children, Toronto, Ontario, Canada; SickKids Inflammatory Bowel Disease Centre, Division of Gastroenterology, Hepatology, and Nutrition, The Hospital for Sick Children, Toronto, Ontario, Canada; Child Health Evaluative Sciences, SickKids Research Institute, The Hospital for Sick Children, Toronto, Ontario, Canada; Department of Medicine, University of Calgary, Calgary, Alberta, Canada; Department of Gastroenterology and Hepatology, Temerty Faculty of Medicine, University of Toronto, Toronto, Ontario, Canada; Department of Neuroscience, McGill University, Montreal, Quebec, Canada; Department of Gastroenterology and Clinical Nutrition, The Royal Children’s Hospital Melbourne, Parkville, Australia; Department of Medicine, University of Calgary, Calgary, Alberta, Canada; Department of Community Health Sciences, University of Calgary, Calgary, Alberta, Canada; Department of Medicine, University of Calgary, Calgary, Alberta, Canada; Department of Community Health Sciences, University of Calgary, Calgary, Alberta, Canada; Department of Medicine, Memorial University of Newfoundland, St John’s Newfoundland, Canada; Department of Interdisciplinary Science, McMaster University, Hamilton, Ontario, Canada; Crohn’s and Colitis Canada, Toronto, Ontario, Canada; Department of Anesthesiology, Pharmacology, and Therapeutics, University of British Columbia, Vancouver, British Colombia, Canada; Crohn’s and Colitis Canada, Toronto, Ontario, Canada; Crohn’s and Colitis Canada, Toronto, Ontario, Canada; Crohn’s and Colitis Canada, Toronto, Ontario, Canada; Division of Gastroenterology and Hepatology, Mount Sinai Hospital, University of Toronto, Toronto, Ontario, Canada

**Keywords:** Crohn’s disease, Biologic, Biosimilar, Management, Therapy, Ulcerative colitis

## Abstract

The therapeutic landscape for inflammatory bowel disease (IBD) has changed considerably over the past two decades, owing to the development and widespread penetration of targeted therapies, including biologics and small molecules. While some conventional treatments continue to have an important role in the management of IBD, treatment of IBD is increasingly moving towards targeted therapies given their greater efficacy and safety in comparison to conventional agents. Early introduction of these therapies—particularly in persons with Crohn’s disease—combining targeted therapies with traditional anti-metabolite immunomodulators and targeting objective markers of disease activity (in addition to symptoms), have been shown to improve health outcomes and will be increasingly adopted over time. The substantially increased costs associated with targeted therapies has led to a ballooning of healthcare expenditure to treat IBD over the past 15 years. The introduction of less expensive biosimilar anti-tumour necrosis factor therapies may bend this cost curve downwards, potentially allowing for more widespread access to these medications. Newer therapies targeting different inflammatory pathways and complementary and alternative therapies (including novel diets) will continue to shape the IBD treatment landscape. More precise use of a growing number of targeted therapies in the right individuals at the right time will help minimize the development of expensive and disabling complications, which has the potential to further reduce costs and improve outcomes.

**Key Points**

1. Over the past two decades, targeted therapies, including biologics and small molecules, have dramatically changed the treatment landscape and improved quality of life for persons with IBD.
2. Strategies to optimize the effectiveness of available therapies, such as introducing biologic therapy early in the course of Crohn’s disease, targeting normalization of objective markers of disease remission, and using therapeutic drug monitoring to guide treatment decisions have the potential to improve long-term prognosis and longevity of current medical treatment options.
3. Randomized controlled trials and real-world studies have demonstrated that biologic therapies are effective and safe for treating IBD. However, proactive use of these therapies early in the disease course and optimizing these therapies based on treatment response along with therapeutic drug monitoring may further improve their effectiveness in clinical practice. Increasing knowledge on how to identify the right therapy for the right person at the right time should further improve long-term outcomes and cost-effectiveness of treatment strategies for persons with IBD.
4. The latest targeted therapies introduced into clinical practice, including selective anti-interleukin-23 inhibitors, sphingosine-1-phosphate agonists, and Janus kinase-1 inhibitors, have shown promising results in randomized controlled trials and may further improve treatment outcomes for persons with IBD.
5. Biosimilar anti-tumour necrosis factor agents have shown similar treatment response rates and safety as their bio-originator counterparts. Biosimilars typically have a substantially lower cost, which may favourably bend the cost curve and promote more widespread access to targeted therapies in clinical practice.
6. Surgery continues to play an important role in IBD management, particularly for stricturing and penetrating complications of Crohn’s disease, perianal fistulizing Crohn’s disease, medically refractory IBD, and intestinal cancers.
7. Evolving therapies directed at modifying gut bacterial flora (e.g., modified diets, probiotics, and faecal microbiota transplant) have shown promise as potential therapies for IBD, although further research is required in these areas before they can be widely recommended.

## INTRODUCTION

The goals of inflammatory bowel disease (IBD) treatment are to induce and maintain disease remission, reduce disease-related complications, prevent permanent bowel damage, and improve quality of life (1). Medical IBD treatment is largely focused on moderating the body’s aberrant immune response to commensal gut bacteria using immunosuppressive therapies. Dietary and alternative medicine treatment strategies, which help modulate inflammation by modifying the gut microbiome, hold promise as therapeutic adjuncts. Additionally, updated goals for IBD treatment and monitoring, including treating to a combined target of sustained bowel healing and elimination of clinical symptoms, may help to improve the long-term IBD course. Despite advances in medical management, surgery continues to play an important role in managing bowel strictures, penetrating complications, perianal disease, and medically refractory intestinal inflammation.

Compared to older non-targeted immunosuppressive therapies, newer classes of IBD treatments, including biologics and small molecules, have shown greater potential to alter the disease trajectory. Newer agents on the horizon will hopefully continue to improve efficacy, safety and tolerability of IBD treatments. The emergence of biosimilar therapies, which have similar effectiveness and safety to bio-originator medications but are typically marketed at a substantially lower cost, may help bend the overall cost curve of biologic therapies downward and improve access to targeted treatments in the future.

In this article, we review current IBD treatments, strategies to optimize available treatments, emerging therapies, and key data supporting current treatment decisions, with a focus on Canadian data where available.

### What Are the Available Medical Treatment Options for People with IBD?

The IBD treatment landscape has evolved rapidly over the last several decades, and the pace of this evolution is increasing (2). Prior to the 1960s, corticosteroids were the only medical therapy available to treat IBD. Five-aminosalicylic acid (5-ASA/mesalamine) and immunomodulators were introduced in the 1960s and 1970s. The first anti-tumour necrosis factor α (anti-TNF) biologic therapy (infliximab) was approved for use in Canada in 2001, followed by a second (adalimumab) in 2004. Since then, several additional biologic therapies and small molecules that target specific inflammatory pathways have been introduced (3). A summary of medications to treat IBD that are approved for use in Canada, their routes of administration, primary indications, and major adverse events is provided in Table 1. In general, biologic and other targeted therapies have demonstrated greater efficacy and improved safety as compared to non-targeted immunosuppressive agents. In particular, approved anti-integrin and anti-interleukin (IL) 12/23/anti-IL 23 agents have achieved a very high standard for safety.

**Table 1.** Currently available treatments for IBD in Canada

Drug	Route of administration	Standard maintenance dose schedule	Treatment phase	Type of IBD	Common side effects and serious adverse reactions (selected)
Topical anti-inflammatory therapies					
5-Aminosalicylates (5-ASA, mesalamine):Older generation (oral) • Sulfasalazine (SSZ) Newer generation (oral) • Pentasa • Salofalk • Mezavant • Octasa • Teva 5-ASA Enemas • Salofalk • Pentasa • Mezera Suppositories • Salofalk • Pentasa	Oral, rectal (suppositories, foams, and enemas)	Oral: Daily Rectal: Daily, though twice weekly has been shown to be beneficial for maintenance of remission	Induction, maintenance	Mild-to-moderate ulcerative colitis (oral); rectal therapy may be used in distal ulcerative colitis. May be beneficial in mild colonic Crohn’s disease, but this is controversial.	Common side effects: • Nausea/vomiting • Paradoxical diarrhoea • Allergic hypersensitivity reactions (causing skin rash and mild fever) • Male infertility (SSZ) Serious adverse reactions (rare): • Serositis (pericarditis, serositis, mesenteritis) • Allergic interstitial nephritis • Cytopenias • Pancreatitis • Hepatitis
Topical corticosteroids: Budesonide (Entocort, Cortiment)	Oral, rectal	Oral: Daily Rectal: Daily	Induction	Mild-to-moderate Crohn’s disease (Entocort) or ulcerative colitis (Cortiment)	See below for corticosteroids N.B. Risk of steroid-related side effects markedly lower than prednisone or solumedrol
Non-targeted immunosuppressive therapies					
Corticosteroids (prednisone, prednisolone, methylprednisolone)	Oral (prednisone) or intravenous (prednisolone, methylprednisolone)	Daily	Induction	Crohn’s disease, ulcerative colitis	Common side effects: • Sleep disturbance • Irritability, mood swings • Mild anxiety or depression • Fluid retention • Increased appetite • Weight gain • Acne • Linear growth delay (children) • Myalgias, arthralgias Serious adverse reactions: • Opportunistic infections • Osteonecrosis • Osteoporosis • Diabetes mellitus • Hypertension • Cataracts • Muscle atrophy • Body fat redistribution (Cushingoid)
Cyclosporine	Oral or intravenous	Daily	Induction	Ulcerative colitis	Common side effects: • Hirsutism • Tremor • GI upset • Headache Serious adverse reactions: • Opportunistic infections • Seizures • Renal toxicity • Hypertension
Thiopurines (azathioprine, 6-mercaptopurine)	Oral	Daily	Maintenance	Crohn’s disease, ulcerative colitis	Common side effects: • GI upset • Hypersensitivity skin and joint reactions Serious adverse reactions: • Opportunistic infections • Pancreatitis • Bone marrow toxicity • Hepatotoxicity • Lymphoma • Non-melanoma skin cancer
Methotrexate	Oral, subcutaneous	Weekly	Maintenance	Crohn’s disease	Common side effects: • Flu-like symptoms • GI upset • Nausea, vomiting • Mucositis Serious adverse reactions: • Bone marrow toxicity • Hepatotoxicity, hepatic fibrosis • Pneumonitis, lung fibrosis
Targeted immuno-active therapies					
Biologics					
Anti-TNFs					
Adalimumab (Humira, Abrilada, Adalimumab injection, Amgevita, Hadlima, Hadlima Pushtouch, Hyrimoz, Hulio, Idacio, Simlandi, Hadlima, Yuflyma)	Subcutaneous	Every two weeks	Induction, maintenance	Crohn’s disease, ulcerative colitis	Common side effects: • Injection site reactions • GI upset • Hypersensitivity reactions (skin, joints) • Upper respiratory tract infections • Headache • Nausea Serious adverse reactions: • Opportunistic infections • Drug-induced lupus • Cardiomyopathy • Demyelinating neuropathy • Lymphoma • Melanoma
Golimumab (Simponi)	Subcutaneous	Every four weeks	Induction, maintenance	Ulcerative colitis	As per Adalimumab
Infliximab (Remicade Inflectra, Ixifi, Renflexis, Remsima, Remsima SC, Avsola)	Intravenous	Every eight weeks	Induction, maintenance	Crohn’s disease, ulcerative colitis	As per Adalimumab Acute infusion reactions (including anaphylaxis)
Anti-integrins					
Vedolizumab (Entyvio)	Intravenous	Every eight weeks	Induction, maintenance	Crohn’s disease, ulcerative colitis	Common side effects: • Acute infusion reactions (IV) • Injection site reactions (SC) • GI upset • Hypersensitivity reactions (skin, joints) • Upper respiratory tract infections • Headache
Anti-IL-12/23s; Anti-IL-23s					
Ustekinumab (Stelara) (Anti-IL-12/23)	Intravenous induction followed by subcutaneous maintenance	Every eight weeks	Induction, maintenance	Crohn’s disease, ulcerative colitis	Common side effects: • Injection site reactions • Upper respiratory tract infections • Headache
Risankizumab (Skyrizi) (Anti-IL-23)	Intravenous induction followed by subcutaneous maintenance	Every eight weeks	Induction, maintenance	Crohn’s disease	Common side effects: • Injection site reactions • Upper respiratory tract infections • Headache
Small molecules					
Janus kinase (JAK) inhibitors					
Tofacitinib (JAK-1/2/3, TYK-2)	Oral	Twice daily	Induction, maintenance	Ulcerative colitis	Common side effects: • GI upset • Hypersensitivity reactions • Upper respiratory tract infections • Headache • Elevated liver enzymes • Hypercholesterolemia Serious adverse reactions: • Opportunistic infections • Herpes Zoster (shingles) • Venous thromboembolism^*^ • Major cardiovascular events^*^ • Cancers^*^
S1P receptor modulators					
Ozanimod	Oral	Daily	Induction, maintenance	Ulcerative colitis	Common side effects: • GI upset • Upper respiratory tract infections • Pyrexia • Headache • Elevated liver enzymes Serious adverse reactions:^†^ • Opportunistic infections • Hypertension • Bradycardia (rare) • Progressive multifocal leukoencephalopathy (rare)

^*^Only demonstrated in studies in rheumatoid arthritis patients.

^†^Until more data available, extrapolated from studies in tofacitinib.

### What Do Large Canadian Studies Tell Us About the Impact of Newer Anti-TNF Therapies on IBD Outcomes?

Most (4–9), but not all (10), Canadian real-world studies have demonstrated declining trends in IBD-related hospitalizations and/or intestinal surgeries in parallel with the introduction of biologic therapies into the marketplace. However, a recent population-based study in Ontario that corrected for secular trends was not able to demonstrate a significant change in the rates of IBD-specific hospitalizations or intestinal surgeries corresponding to the period following marketplace introduction of infliximab among persons with Crohn’s disease over a 10-year period or among persons with ulcerative colitis over five-year period; this suggests that factors other than anti-TNF therapy may have also contributed to the observed trends in earlier studies (7). Data from Ontario and Manitoba have also shown increasing penetration of anti-TNF therapy in persons with Crohn’s disease over the first decade following market introduction, but very little uptake in individuals with ulcerative colitis; this suggests that underuse of biologic therapies may limit the population-level impact on ulcerative colitis disease course (11, 12). An ongoing multi-provincial study by the Canadian Gasto-Intestinal Epidemiology Consortium with longer follow-up aims to further evaluate the impact of more widespread uptake of biologic and other targeted therapies on IBD outcomes across Canada.

A population-based study from Manitoba further showed that initiation of anti-TNF treatment in the first two years following a diagnosis of Crohn’s disease was associated with 4.5 fewer IBD-specific hospitalizations (95% CI: 2.10), and 10.4 fewer all-cause hospitalizations (95% CI: 3.7, 17.0) per 100 person-years, over the five years following the start of therapy (13). The decrease in IBD-specific and all-cause hospitalizations was most prominent in the latter half of the five-year follow-up period. The adjusted cumulative surgery rate over the five years after beginning anti-TNF therapy was not significantly different between those who began the therapy early or late in the follow-up period (5.7 vs 7.3 operations per 100 person-years; risk difference, −1.6 [95% CI, −4.5, 1.3]). However, when the first year of follow-up after starting anti-TNF therapy is excluded, early anti-TNF therapy was associated with 3.6 fewer surgeries per 100 person-years (95% CI, 1.9, 5.3). Similarly, data from a multicentre study in 552 persons younger than age 17 diagnosed with inflammatory (non-penetrating, nonstricturing) Crohn’s disease between 2008 and 2012 at 28 paediatric gastroenterology centres in North America found that treatment with anti-TNFα therapy within three months of diagnosis was superior to early treatment with an immunomodulator alone (85.3% vs. 60.3% in remission; relative risk: 1.41; 95% CI: 1.14 to 1.75; *p* = 0.002) in achieving clinical remission at one year (14). A landmark pragmatic randomized controlled trial (RCT) conducted in Belgian and Canadian non-academic centres showed that early introduction of combined immunosuppression with an anti-TNF agent and an anti-metabolite immunomodulator in persons with Crohn’s disease reduced the risk of surgery, hospital admission, and/or serious disease-related complications at 24 months as compared to conventional step-up therapy (27.7% and 35.1%, absolute difference: 7.3%, hazard ratio: 0.73; 95% CI: 0.62 to 0.86; *p* < 0.001) (15).

A population-based study from Manitoba further showed an annual reduction in corticosteroid use of 3.8% over the past two decades in persons with Crohn’s disease (most marked after 2007) and of 2.5% in persons with ulcerative colitis, which could relate to increasing penetration of biologic therapies and greater recognition of the potential adverse events associated with of long-term corticosteroid use (16). Similar findings were observed in a population-based study from Alberta, with an average annual decline in corticosteroid use of more than 18% among persons with IBD between 2010 and 2015, coinciding with increasing penetration of anti-TNF therapy (17).

Given the relatively recent introduction of other classes of biologic and targeted therapies, limited real-world Canadian data exist for these therapies. Such studies will hopefully inform a future review of the IBD treatment landscape.

## HOW DO WE OPTIMALLY USE IBD TREATMENTS TO IMPROVE LONG-TERM DISEASE OUTCOMES?

Several strategies have emerged over the past 10 to 15 years that have improved our ability to optimize the effectiveness of targeted therapies used to treat IBD.

### Treat-to-Target

**Figure 1.**
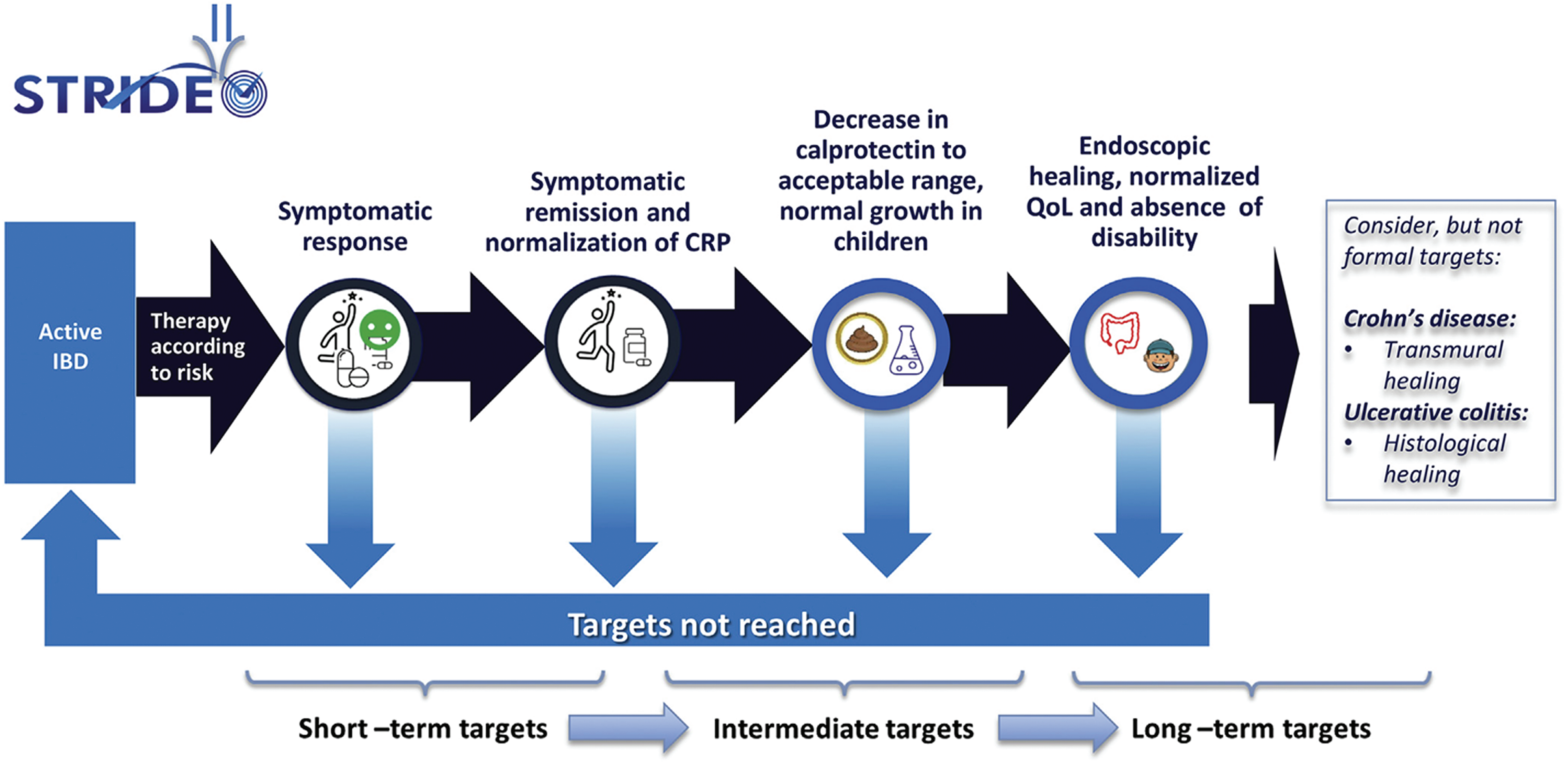
STRIDE-II.

Selecting Therapeutic Targets in Inflammatory Bowel Disease-II (STRIDE-II) was a landmark paper guiding treatment targets in adults and children with IBD (1). In those with IBD experiencing a flare of disease activity, the short-to-intermediate-term goal is to achieve rapid symptomatic response followed by clinical remission and normalization of C-reactive protein (CRP) and faecal calprotectin. The longer-term targets include endoscopic healing, normalization of growth (in children), normal quality of life, and absence of disability (Figure 1). Specific timelines for meeting these targets were not provided in the STRIDE-II document as they may vary across disease phenotypes and treatment agents. In general, short term may be considered as four to six weeks, intermediate term as three to six months and longer term as six to 12 months and beyond. When these targets are not met, a new strategy of managing IBD is required, such as optimizing the dose of therapy, using adjuvant therapies, changing medications, or surgery.

Achieving treat-to-target (T2T) goals requires regular disease activity surveillance that evaluates a combination of symptoms, blood work, faecal calprotectin, bowel imaging, and endoscopy. The most notable innovation has been targeting objective markers of disease remission, particularly faecal calprotectin and endoscopic and radiographic healing, in combination with reducing symptoms (18–21). Importantly, gastrointestinal symptoms that persist despite achieving objective disease remission are common and may relate to chronic bowel damage or superimposed irritable bowel syndrome that does not require treatments that target IBD (22).

Multiple studies have demonstrated the value of endoscopic healing on long-term IBD prognosis (23–26). A T2T strategy has further been shown to increase the probability of endoscopic healing (18,21). An RCT, the effect of tight control management on Crohn’s disease (CALM), demonstrated that adjusting medical management based on regular disease activity surveillance using symptoms and biomarkers, such as CRP and faecal calprotectin, improves clinical and endoscopic outcomes out to three-years (21). A follow-up Canadian study further demonstrated that this treatment approach is cost-effective relative to symptom-based management (27).

### Therapeutic Drug Monitoring

Therapeutic drug monitoring (TDM) of biologic agents has permitted a more scientific method to guide treatment decisions. Such monitoring is most relevant for the anti-TNF class of therapy, as the chimeric nature of the molecules leads to greater potential for the development of anti-drug antibodies and permanent loss of efficacy (28). In the setting of on-going or recurrent disease activity during treatment with a biological agent, the presence of high plasma levels of drug or anti-drug antibodies indicates either a mechanistic switch of disease activity (to another immune phenotype) or the development of irreversible immunogenicity, associated with markedly reduced drug effectiveness and the need to switch to another agent (29,30). Conversely, the presence of low circulating levels of drug or of anti-drug antibodies in the setting of a disease flare may be managed, at least in the short term, through optimizing drug dosage (29,30). An expert international panel unanimously voted a reactive TDM strategy should be used for all biologics to help manage both primary non-response and secondary loss of response (30); this approach has been demonstrated to be cost-effective relative to empiric drug optimization (31). Furthermore, it was recommended that treatment discontinuation should not be considered for infliximab or adalimumab until a drug concentration of at least 10–15 μg/mL is achieved (30). Importantly, the target drug concentration to effect disease activity may vary depending on the disease phenotype, the assay used, and the desired therapeutic outcome; thus, treatment decisions based on TDM should be individualized (30). Some individuals may require higher-than-established drug concentrations to establish optimal long-term disease control, such as individuals with perianal fistulizing Crohn’s disease or small bowel Crohn’s disease (32).

Proactive TDM, a strategy in which biologic treatment escalation or de-escalation is guided purely by plasma drug and/or anti-drug antibody levels, in the absence of objective disease activity, has not demonstrated convincing evidence of benefit with respect to health outcomes and is associated with a substantially higher cost than conventional management (33). However, a large RCT demonstrated that proactive dose adjustment of adalimumab when treating paediatric Crohn’s disease was associated with a higher rate of corticosteroid-free clinical remission, a higher rate of composite sustained corticosteroid-free clinical remission, normal CRP, and normal faecal calprotectin at all visits from weeks eight to 72 when compared to reactive TDM (34). The personalized anti-TNF therapy in Crohn’s disease study, a large prospective UK study in anti-TNF-naïve people aged ≥6 years with active luminal Crohn’s disease at first exposure to infliximab (*n* = 955) or adalimumab (*n* = 655), found that week 14 drug concentration was independently associated with the probability of week 14 primary non-response (infliximab, odds ratio [OR]: 0.35; 95% CI: 0.20, 0.62; adalimumab, OR: 0.13; 95% CI: 0.06, 0.28); week 54 non-remission (infliximab, OR: 0.29; 95% CI: 0.16, 0.52; adalimumab, OR: 0.03; 95% CI: 0.01, 0.12) and the development of anti-drug antibodies (infliximab: 62.8%; 95% CI: 59.0, 66.3; adalimumab: 28.5%; 95% CI: 24.0, 32.7) (35). This study further reported that the optimal week 14 drug concentrations associated with remission at both week 14 and week 54 were 7 mg/L for infliximab and 12 mg/L for adalimumab. In light of these data, an international panel voted strongly in favour of proactive TDM once post-induction, at least once during maintenance therapy, after any reactive TDM-based treatment change, and before and after any dose escalation or de-escalation to guide drug dosing of anti-TNF therapy; the panel noted the need for more data to support proactive TDM for other biologic agents (30).

### Step-up Versus Top-down Approaches to Therapy With Anti-TNFs

Multiple RCTs have demonstrated that early combined immunosuppression of an anti-TNF and a thiopurine early in the disease course can improve disease outcomes compared with a conventional step-up treatment strategy or anti-TNF monotherapy (15, 36–38). Population-level data from Manitoba and other Canadian provinces have similarly demonstrated a positive impact of early combined immunosuppression on the risk of future IBD-related complications (39,40), as well as earlier introduction of anti-TNF therapy on healthcare utilization in persons with Crohn’s disease (13). The long-term impact on colectomy of early medical intervention for individuals with ulcerative colitis is less clear. At present, the considerably higher costs of targeted therapies as compared to conventional agents limits access to early combined immunosuppression for individuals dependent on public drug coverage. Fortunately, some provinces have recently approved access to biosimilar anti-TNF agents and vedolizumab without the requirement for loss of effectiveness of an immunomodulator.

### Choosing the Right Therapy for the Right Person at the Right Time

Given the increasing variety of drugs available to treat IBD, there is great interest in understanding how to choose the right drug for the right person at the right time. To date, only two RCTs have directly compared approved targeted therapies for IBD. In the VARSITY trial, vedolizumab was superior to adalimumab at standard dosing for achieving clinical remission (although not steroid-free remission) in persons with moderate-to-severe ulcerative colitis (41). In the SEAVUE trial, ustekinumab was equivalent to adalimumab in attaining symptomatic and endoscopic remission in persons with Crohn’s disease. While there are other head-to-head trials ongoing, these will only assess a fraction of the possible comparisons that can potentially be made between drugs. Network meta-analyses (NMA) have tried to fill in some of these data gaps through indirect comparisons. Recent NMAs have suggested that anti-TNFs and anti-IL-23 therapies may be superior to other therapies for Crohn’s disease, and anti-TNF (infliximab), anti-IL-23, and Janus kinase (JAK) inhibitor therapies (tofacitinib) may be most effective for ulcerative colitis, while anti-integrin therapy (vedolizumab) ranks highest for safety (42–44). Importantly, differences in the design and populations of studies included in NMAs may account for some of the apparent differences. Further research will be required to identify which specific sequence or combination of available agents, possibly in conjunction with other strategies of medical treatment (such as modulation of gut bacteria), optimally impacts long-term disease prognosis in individuals.

## HOW WILL BIOSIMILAR AGENTS IMPACT TREATMENT OPTIONS FOR CANADIANS WITH IBD?

A biosimilar medication must show a high degree of similarity to the original product and have no clinically meaningful differences in safety, purity, or potency (45). As several anti-TNF bio-originator compounds have now exhausted their patency, multiple biosimilar anti-TNF agents have been developed and are now commercially available, often at lower prices than their originator molecules. Given that spending on biologic medications is currently responsible for the majority of direct healthcare spending in IBD (see Kuenzig et al., this volume), the availability of less expensive alternatives could yield substantial cost savings. As a result, many provinces and insurers have been instituting policies that favour the use of biosimilar formulations of infliximab and adalimumab in place of their bio-originator forms (Remicade and Humira, respectively). In addition, six Canadian provinces have either instituted or have imminent plans to institute a mandatory switch policy, whereby the majority of current users of Remicade and Humira will be required to switch to a corresponding biosimilar to maintain insurance coverage.

Most studies comparing bio-originator to biosimilar anti-TNF therapies for anti-TNF-naïve individuals have not shown any meaningful differences in objective IBD-related or safety outcomes (46–48). A systematic review of clinical trials that investigated biosimilar infliximab in anti-TNF-naïve people with IBD and in people with IBD who switched from originator infliximab also did not show any significant differences in efficacy or safety between the originator infliximab and its biosimilar. However, a recent systematic review and position statement released by the Canadian Association of Gastroenterology and Crohn’s and Colitis Canada reported that very low-quality evidence, according to GRADE criteria, does not support nonmedical switching to biosimilar infliximab in persons who have stable IBD and are doing well on the originator product due to an increased risk of worsening disease, dose escalation, and/or switching to an alternative. (45). This position is shared by some, but not all, national societies. Nonetheless, given the potential cost savings and minimal risk of disease recurrence associated with non-medical switching, most North American and European societies, concur that this is an acceptable approach if agreed upon by both the physician and the individual (45,49–53). Conversely, the overwhelming majority of societies do not support the mandatory substitution of a biosimilar agent for the originator agent in all individuals due to a paucity of evidence for the efficacy and safety of this approach (45,49). Crohn’s and Colitis Canada has suggested the use of a risk matrix (https://crohnsandcolitis.ca/Get-Involved/Advocating-for-change/Non-Medical-Switch-Biosimilars) to guide biosimilar switching in persons with IBD. The position statement by the Canadian Association of Gastroenterology and Crohn’s and Colitis Canada did provide a weak recommendation that an infliximab biosimilar could be started in people with active Crohn’s disease who are naïve to anti-TNF therapy, for cost reasons, but noted that there were insufficient data to recommend the use of biosimilars in people with active ulcerative colitis naïve to infliximab.

Importantly, it has been reported that 10%–20% of individuals may experience a nocebo effect with biosimilar switching (an increase in symptoms that follow a perception of a change in therapy) (54,55), particularly those who have high levels of anxiety and a tendency toward catastrophization (56). The impact of nocebo effects can be mitigated through involvement of the individual in the decision-making process, setting expectations of a positive outcome, and identification of persons in who may be at higher risks for developing nocebo effects (54).

While more data are needed, in the absence of a significant observable deterioration in individual health outcomes in most studies to date evaluating biosimilar start or switch, biosimilar agents offer an attractive solution to the ballooning costs associated with IBD therapies. What is less clear is to what extent these cost savings will be re-invested to the betterment of IBD care and research. At a minimum, the lower costs associated with biosimilar agents, alongside the parallel lowering of costs of their bio-originator counterparts, should allow for improved access to these agents. Several provincial drug formularies have now agreed to fund biosimilar anti-TNF therapies for individuals with IBD irrespective of prior failure of conventional antimetabolite immunomodulating agents, which is not the case for most bio-originator drugs.

## WHAT’S ON THE HORIZON FOR IBD TREATMENT?

In the last year, two new drugs were approved by Health Canada for the treatment of IBD. Risankizumab (Skyrizi) was approved for the treatment of moderate-to-severe Crohn’s disease, and ozanimod (Zeposia) was approved for the treatment of moderate-to-severe ulcerative colitis. Upadacitinib (Rinvoq) is also being considered for approval in Canada.

Risankizumab is the first drug that specifically targets IL-23 approved for Crohn’s disease (57,58). It has been approved in Canada since 2019 for psoriatic arthritis and plaque psoriasis, and, thus far, has not demonstrated any increased risk of serious complications in comparison to other medications (59). A head-to-head trial comparing risankizumab to ustekinumab in Crohn’s disease is underway (60). A head-to-head trial of these agents in persons with plaque psoriasis has demonstrated superiority of risankizumab over ustekinumab (61).

Ozanimod is an oral sphingosine-1-phosphate (S1P) inhibitor, and is the first drug in its class approved in Canada for moderate-to-severe ulcerative colitis (62). It has been approved in Canada since 2017 for multiple sclerosis and has been shown to have an excellent safety profile over the long-term in this condition (63,64).

Upadacitinib is a JAK-1-specific inhibitor that, in its initial trials, has yielded remission and response rates higher than those seen with other drugs (65). Though there have not been head-to-head comparisons, indirect comparisons with other therapies suggest that upadacitinib may be one of the more effective therapies currently available for ulcerative colitis (66). Concerns have been raised about JAK inhibitor users being at higher risk of cardiovascular, cancerous, and thromboembolic complications based on studies evaluating another JAK-inhibitor (tofacitinib) in elderly persons with rheumatoid arthritis (67). The only serious adverse event that has been consistently associated with this class of medications in persons with ulcerative colitis is herpes zoster (i.e., shingles), typically with higher dose, long-term usage (68). However, this risk is also elevated in people using anti-TNFs and may be reduced through vaccination against herpes zoster virus, as suggested for all adults with IBD on immunosuppression by clinical practice guidelines from the Canadian Association of Gastroenterology (69).

Agents that currently have active research programs in IBD and may become available in coming years include other selective JAK inhibitors (filgotinib for ulcerative colitis and Crohn’s disease (70,71), upadacitinib for Crohn’s disease (72)), other selective IL-23 inhibitors (mirikizumab and guselkumab for Crohn’s disease and ulcerative colitis (73,74)), other S1P receptor modulators (ozanimod for Crohn’s disease, etrasimod for ulcerative colitis (75)) and mesenchymal stem cell treatment for perianal fistulizing Crohn’s disease (76). Health Canada approval of these agents will depend on their efficacy and safety in ongoing clinical trials, and public drug coverage will depend on the ability to demonstrate that these agents will achieve meaningful improvements in the quality of life for Canadians with IBD at a reasonable cost. Most likely, these agents will initially be funded as second or third-line agents in public drug benefits programs across Canada. Crohn’s and Colitis Canada will continue to advocate for Canadians living with IBD to receive the best available treatments when they are required.

## WHAT IS THE ROLE OF ALTERNATIVE AND MICROBIOME-ALTERING THERAPIES IN IBD CARE?

The application of treatments that are neither Health Canada-approved immune-modulating medications nor surgery in the management of IBD, known as complementary and alternative medicine (CAM), are common among persons with IBD. The prevalence of CAM use has been estimated to be as high as 50% in some studies (77–80). There are many potential contributors to the use of CAM in these individuals, including lack of efficacy of conventional therapies, safety concerns with conventional therapies, and improved sense of control over the disease (79). Despite the high prevalence of CAM use, there is limited data demonstrating its efficacy in the treatment of IBD. In recent years, however, there has been emerging data evaluating microbiome-altering therapies such as faecal microbiota transplantation (FMT) and probiotics.

The composition of the gut microbiome has been shown to have a significant influence on the body’s immune response, and changes in the microbiome have been implicated in the development of IBD and flares (81). As a result, there is great interest in emerging therapies that seek to restore a healthy microbiome, such as FMT and probiotics, in the hopes that this will decrease intestinal inflammation and reduce symptom burden. In FMT, faecal material from a healthy individual is introduced via enema, colonoscopy, or nasogastric tube into the intestine of someone with IBD; the aim is to supplant the microbiome of the recipient with that of the healthy donor. A recent meta-analysis of six RCTs found that FMT was associated with higher odds of clinical and endoscopic remission as compared to placebo in persons with ulcerative colitis (OR: 4.11; 95% CI: 2.19, 7.72), with no difference in the risk of side effects (82). A more recent study of 66 people with ulcerative colitis in clinical remission who were randomized to either the combination of FMT and an anti-inflammatory diet or standard medical therapy noted higher rates of clinical and endoscopic remission at 48 weeks (25% vs. 0%, *p* = 0.007) in the FMT arm (83). There is less evidence supporting the role of FMT in maintenance of remission in ulcerative colitis or in the treatment of Crohn’s disease. Two RCTs evaluating the role of FMT in Crohn’s disease have shown improvements in short-term clinical remission rates (84,85). Most other studies evaluating the role of FMT in Crohn’s disease are limited by small study size, heterogeneity, and publication bias. Though the FMT data is promising in ulcerative colitis, it is still not available as a therapeutic strategy for IBD outside of clinical trials.

Probiotics, defined as products containing specific strains of live microorganisms that can be taken orally, are also commonly used by individuals with IBD, despite a lack of convincing evidence on effectiveness or safety. The American Gastroenterology Association recently issued Clinical Practice Guidelines stating that there is no evidence of benefit to any probiotic for either induction or maintenance of remission and suggested that probiotics should only be used in the context of clinical trials. This document did make a conditional recommendation for a specific 8-strain probiotic combination in the treatment of individuals with pouchitis based on a review of seven studies (86), four of which supported a role of this probiotic in the prevention of pouchitis flares (87–90). The quality of evidence was rated as very low.

## WHAT IS THE ROLE OF DIET IN IBD?

Many people look to dietary therapy as either an alternative or an adjuvant treatment to conventional IBD management. To date, the strongest evidence to support dietary therapy is in paediatric IBD. Exclusive enteral nutrition (EEN), whereby individuals receive all nutritional intake through a formula for up to 12 weeks delivered orally, via nasogastric tube, or a gastrostomy tube, has been shown to be effective in induction of remission in Crohn’s disease (91,92). While this treatment requires a significant commitment from both the individual and their caregivers and adherence can be a challenge, some guidelines favour it over corticosteroids, particularly in children with a history of delayed growth (93,94). Partial enteral nutrition is another nutritional therapy that may be better tolerated than EEN. A 2019 study evaluated a combination of partial enteral nutrition with a specific whole foods-based exclusion diet (the Crohn’s disease exclusion diet [CDED]) (95). Participants were randomized to receive partial enteral nutrition and the CDED for 12 weeks or EEN for six weeks, then transition to partial enteral nutrition and a free diet. The CDED consists of a diet that avoided or reduced exposure to foods containing animal/dairy fat, high fat from other sources, wheat, red or processed meat and protein sources rich in taurine, emulsifiers, artificial sweeteners, carrageenans, and sulphites. The second phase step-down diet involves higher exposure to fruits, vegetables, and legumes along with some foods that are reintroduced with restrictions to increase food flexibility and relieve monotony. Among 74 participants at week six, 75% of children given CDED plus partial enteral nutrition were in corticosteroid-free remission versus 59% given EEN. At week 12, 75.6% of children given CDED plus partial enteral nutrition were in corticosteroid-free remission versus 45.1% given EEN. Partial nutrition and CDED were also better tolerated than EEN.

The data to support of the use of dietary therapy in adults with IBD is not as strong. A Cochrane systematic review on diet for induction of remission in Crohn’s disease concluded that all studies provided low or very low-quality evidence (96). One of the most common therapeutic diets used by adults is the Specific Carbohydrate Diet (SCD) (97). The Mediterranean diet has also become increasingly popular and several studies have identified a lower risk of Crohn’s disease among populations consuming this diet, which consists of fruits, vegetables, nuts, fish, whole grains, and use of olive oil as the predominant fat source (98). A recent study compared these two diets (99). Adults with Crohn’s disease were randomly assigned 1:1 to consume the Mediterranean or SCD for 12 weeks. The primary outcome was symptomatic remission at week six. Among the 194 participants, SCD was not superior to Mediterranean diet to achieve symptomatic remission, faecal calprotectin response, or CRP response. The authors concluded that the greater ease of following the Mediterranean diet and its other health benefits make it preferred to the SCD for most individuals with Crohn’s disease.

## WHAT IS THE ROLE OF SURGERY IN IBD MANAGEMENT?

Surgery, once the mainstay of IBD treatment, continues to play an important role in the management of IBD due to failure or inadequate usage of medical therapy or the development of disease-related complications. Often, surgery complements medical therapy, particularly for perianal fistulizing disease and fibrostenotic small bowel disease. Common reasons for IBD surgery include perianal abscesses or persistently draining fistula tracts, fibrotic intestinal strictures resulting in bowel obstruction, penetrating intestinal complications (such as intra-abdominal abscess or enteric fistulas), treatment-refractory disease resulting in persistent and/or rapidly escalating disease activity (sometimes associated with complications such as toxic megacolon or bowel perforation), and intestinal cancer. A multicenter RCT from the Netherlands and the United Kingdom in persons with non-stricturing ileocecal Crohn’s disease affecting <40 cm of small bowel, in whom conventional therapy had failed, demonstrated that surgical resection was associated with similar health outcomes and quality of life as infliximab treatment and was more cost-effective in persons with limited small bowel Crohn’s disease (100,101). Close collaboration between medical and surgical IBD specialists is important for the management of complex IBD phenotypes.

## CONCLUSION

The IBD therapeutic landscape and treatment goals have changed dramatically over the past two decades, and we can anticipate further changes in the years to come as more drugs with different mechanisms of action and a greater number of biosimilar agents gain Health Canada approval. An improved understanding of matching drugs and treatment strategies to individuals may lead to better health outcomes, prevent complications, and improve quality of life.

## KNOWLEDGE GAPS AND FUTURE RESEARCH DIRECTIONS

1. Understanding the factors that may predict individual-level response to drugs with specific mechanisms of action will allow physicians to choose the right therapies for the right individual at the right time.
2. Understanding the changes in the immune system that lead to loss of response to a previously effective medication may help mitigate loss of effectiveness or change the choice of individual therapy.
3. RCTs and real-world evidence to understand the comparative effectiveness of different types and combinations of medical therapies in persons with specific IBD subtypes, as well as pragmatic trials and real-world evidence to better understand how to optimize the usage and sequencing of medical therapies in clinical practice will better enable T2T and personalized treatment strategies.
4. Real-world evidence is required to inform the effectiveness and safety of newer biosimilar agents that are entering the marketplace.
5. Future clinical trials and observational studies of IBD therapies should aim to include under-represented populations, such as Indigenous peoples, pregnant people, paediatrics, seniors, and immigrants.

## PATIENT AND CAREGIVER PARTNER PERSPECTIVE

This article provided patient partners with hope and peace of mind particularly related to the ongoing research towards developing new medication options to treat IBD. Partners were also reassured from the research related to the safety, and efficacy of biosimilars as more provinces enforce a non-medical switch from biologics. The availability of biosimilars can enhance access to life-changing medications for persons with IBD. Patient partners encouraged greater education for patients and caregivers related to the safety and efficacy of switching from biologic to biosimilar medications to decrease anxiety related to non-medical switching. Partners encouraged individuals with IBD who are non-medically switching to biosimilar medications to maintain positive thoughts about the switch to prevent nocebo effects. Selecting the right therapy for the right person at the right time was seen as an important strategy to adopt. Also, combination therapies for certain individuals could result in better outcomes. Partners recognized that complementary therapies have potential roles as treatment adjuncts, particularly in individuals living with ulcerative colitis.

## POLICY IMPLICATIONS AND KEY ADVOCACY OUTCOMES

1. All Health Canada-approved therapies to treat IBD should be accessible to individuals when deemed necessary to control their disease by prescribing physicians. Advocacy should target barriers to accessing the safest and most effective medications as part of shared decision-making between the individual and the physician.
2. Increasing acceptance of biosimilar agents by individuals with IBD and their practitioners should be encouraged to help control the rate of rising drug costs to treat IBD, enhance competitive pricing of biotherapies and improve access to biotherapies.
3. Non-medical switch policies should consider the patient experience and integrate both the risk of increased disease activity and the impact of disease on the individual and family. Use of Crohn's and Colitis Canada' Risk Matrix to help guide non-medical switch policies implemented by provincial health ministries is encouraged. Vigilance should be maintained to ensure that new biosimilar agents that enter the marketplace are held to the same standards for efficacy and safety as their bio-originator counterparts.
4. Healthcare practitioners and afflicted individuals should be informed of the nocebo effect and tools (including mental health support) should be introduced to help mitigate it in those forced to switch.
5. The cost savings realized from increasing use of biosimilar agents should be redirected towards improving access to targeted therapies and diagnostic testing for persons with IBD and increasing research funding for IBD.
6. Tools required to monitor individuals according to a T2T approach, including, but not limited to endoscopy, cross-sectional imaging, and faecal calprotectin should be readily available to practitioners to assist with IBD management.
7. Scientific literature on the evidence for IBD treatment options should be free and accessible to all Canadians to better inform individuals and health care providers as to the options and modifiable behaviours for disease ­control.

## SUPPLEMENT SPONSORSHIP

This article appears as part of the supplement “The Impact of Inflammatory Bowel Disease in Canada in 2023”, sponsored by Crohn’s and Colitis Canada, and supported by Canadian Institutes of Health Research Project Scheme Operating Grant (Reference number PJT-162393).

## Data Availability

No new data were generated or analyzed in support of this review.
